# Ancillary Tests for Death by Neurologic Criteria in Children

**DOI:** 10.15844/pedneurbriefs-34-26

**Published:** 2020-12-24

**Authors:** Elizabeth Mayne, Sue J. Hong

**Affiliations:** 1 Divisions of Neurology and Critical Care Medicine, Anne and Robert H. Lurie Children's Hospital of Chicago, Chicago, IL

**Keywords:** Brain Death, Death by Neurologic Criteria, Ancillary Test, EEG, Pediatrics, Neurology

## Abstract

Investigators from Children's Hospital of Pennsylvania reported on the usage of ancillary studies in the declaration of brain death in children in a single-center retrospective descriptive study.

Investigators from Children's Hospital of Pennsylvania reported on the usage of ancillary studies in the declaration of brain death in children in a single-center retrospective descriptive study. In their cohort of 73 patients, 47% underwent ancillary testing. Nearly all of those patients (88%) had a technetium brain scan; the remaining 12% (4/34) had an electroencephalogram (EEG). Nearly half of the ancillary tests (16/34) were performed because of the inability to complete the clinical exam, primarily due to cervical spine injuries (10/16). Other common indications included the inability to perform the apnea test (15% of ancillary tests) and uncertainty about the neurologic exam (12%). Only one of the 34 ancillary tests was not consistent with brain death; this result occurred in a child with a devastating neurologic injury who subsequently had two complete clinical exams and apnea tests that were consistent with death by neurologic criteria (DNC). [1]

COMMENTARY. The 2011 AAP guidelines for declaration of DNC include two separate clinical exams and apnea tests consistent with brain death, separated by 12-24 hours, depending on the patient's age [2]. Ancillary studies, which can include EEG and technetium cerebral blood flow study, are not required unless: an element of the clinical exam or apnea testing cannot be completed, there is uncertainty about the results of the neurologic exam, a medication effect may confound the exam, or reduction of the inter-examination period is desired. Despite guidance regarding ancillary testing in these specific scenarios, there is wide variability in institutional protocols and individual practice regarding the use of ancillary tests in DNC declaration and which ancillary studies are available [3]. In a recent survey of US pediatric intensivists and neurologists, 20% perform ancillary tests for reasons beyond those outlined in the national guidelines, with some respondents routinely incorporating an ancillary study [4]. In this study, most tests (79%) were performed according to the current DNC guidelines. Other indications were performed by parental request, three were performed based on institutional protocol more stringent than national guidelines, and three were performed with indications unknown. No other patients in this cohort had ancillary testing indications that did not subsequently have ancillary testing performed.

The high rate of ancillary testing in this cohort reflects the challenges and uncertainties of DNC clinical evaluation in pediatric populations. However, ancillary studies have low sensitivity and specificity. They require providers with expertise in performing these tests and interpreting the results in the context of a DNC declaration in a pediatric patient. The high rate of congruence between the clinical exam and the ancillary studies results in this study highlight the importance of the clinical exam in determining an appropriate patient population in whom the pre-test probability of a positive test is high [1]. Ancillary studies are best used when necessary and as confirmatory, rather than diagnostic, tests. DNC is a clinical diagnosis; however, the high prevalence of indications for ancillary testing in this study also highlights a pressing need to ensure that institutions performing DNC evaluations in children are competent in the indications, techniques, and interpretations of ancillary testing for brain death.

## Disclosures

The authors have declared that no competing interests exist.
